# Factors Influencing Adherence to Antiretroviral Treatment in Nepal: A Mixed-Methods Study

**DOI:** 10.1371/journal.pone.0035547

**Published:** 2012-05-01

**Authors:** Sharada P. Wasti, Padam Simkhada, Julian Randall, Jennifer V. Freeman, Edwin van Teijlingen

**Affiliations:** 1 School of Health and Related Research (ScHARR), University of Sheffield, Sheffield, United Kingdom; 2 Business School, University of Aberdeen, Aberdeen, United Kingdom; 3 Health and Social Care, Bournemouth University, Bournemouth, United Kingdom; University of Ottawa, Canada

## Abstract

**Background:**

Antiretroviral therapy (ART) is a lifesaver for individual patients treated for Human Immunodeficiency Virus (HIV) and Acquired Immune Deficiency Syndrome (AIDS). Maintaining optimal adherence to antiretroviral drugs is essential for HIV infection management. This study aimed to understand the factors influencing adherence amongst ART-prescribed patients and care providers in Nepal.

**Methods:**

A cross-sectional mixed-methods study surveying 330 ART-prescribed patients and 34 in-depth interviews with three different types of stakeholders: patients, care providers, and key people at policy level. Adherence was assessed through survey self-reporting and during the interviews. A multivariate logistic regression model was used to identify factors associated with adherence, supplemented with a thematic analysis of the interview transcripts.

**Results:**

A total of 282 (85.5%) respondents reported complete adherence, i.e. no missed doses in the four-weeks prior to interview. Major factors influencing adherence were: non-disclosure of HIV status (OR = 17.99, p =  0.014); alcohol use (OR = 12.89, p = <0.001), being female (OR = 6.91, p = 0.001), being illiterate (OR = 4.58, p = 0.015), side-effects (OR = 6.04, p = 0.025), ART started ≤24 months (OR = 3.18, p = 0.009), travel time to hospital >1 hour (OR = 2.84, p = 0.035). Similarly, lack of knowledge and negative perception towards ART medications also significantly affected non-adherence. Transport costs (for repeat prescription), followed by pills running out, not wanting others to notice, side-effects, and being busy were the most common reasons for non-adherence. The interviews also revealed religious or ritual obstacles, stigma and discrimination, ART-associated costs, transport problems, lack of support, and side-effects as contributing to non-adherence.

**Conclusion:**

Improving adherence requires a supportive environment; accessible treatment; clear instructions about regimens; and regimens tailored to individual patients’ lifestyles. Healthcare workers should address some of the practical and cultural issues around ART medicine whilst policy-makers should develop appropriate social policy to promote adherence among ART-prescribed patients.

## Introduction

The advent of antiretroviral treatment (ART) has dramatically slowed down the progression of HIV, reduced the death rate from AIDS and transformed the infection from a fatal illness to a more manageable chronic illness [1], [2]. Since 2004, Nepal has been providing free-of-cost ART and by the end of 2009, over 2,524 adults received free ART at 23 sites across the country out of an estimated over 63,528 People Living with HIV (PLHIV) nationally [3]. The success of a national scale-up of ART depends on bolstering the capacity of the health care system and shifting its orientation from acute care to a chronic-care model [4], [5].

However, simply making ART medicine available to PLHIV is not enough, as strict adherence is required for treatment success [2], [6]. Poor adherence can lead to the virological failure of cheap first-line treatment regimens and the spread of multi-drug resistant forms of the virus, resulting in a public health calamity [2], [7], [8]. Unlike many other diseases, it is vital that PLHIV consume all doses of the drug to prevent resistance and to improve their chances of survival. Understanding the level of non-adherence and the factors that lead to it are important clinical and public health goals. This information is essential to inform ART programmes and maximise the success of treatment.

Paterson and colleagues found that adherence greater than 95% is needed to achieve virological success; however 22% of patients with an adherence level of over 95% experienced virological failure (i.e. a sharp increase in viral load) compared to 61% of patients with adherence between 80–94.9%, and 80% of patients with an adherence level of below 80% [2]. A meta-analysis of studies of ART adherence found that a pooled estimate of 77% of patients in Africa achieved adequate adherence (>95% of prescribed pills) compared to just 55% of patients in North America [9]. However, the relationship between adherence and the development of resistance differs by regimens; for example resistance to non-nucleoside reverse transcriptase inhibitors is significantly higher at low levels of adherence than that for protease inhibitors [10].

Prescribers hope that every patient completely follows their ART instructions, but the literature shows that a proportion of PLHIV do not take medications as prescribed for various reasons. A patient’s ability to adhere to medication is greatly influenced by both individual and environmental factors. Several studies have shed light on the factors affecting adherence, highlighting socio-demographic, cultural, economic, health-systems and treatment-related factors [9], [11], [12]. Many barriers to adherence are common to both developed and developing countries such as fear of disclosure [9]. However, some are more common in the Asian developing countries such as ART-associated costs (transport fares, diagnostic costs) and problems with travel to access treatment [13]. Hence, to benefit from ART, it is important to identify adherence behaviour, understand the conditions that lead to non-adherence and develop strategies and social policies to maximise long-term adherence. This study was designed to identify the current levels of adherence and the factors influencing adherence to ART in Nepal, as to our knowledge, there is no prior study of this kind in Nepal.

## Methods

A cross-sectional mixed-methods study was conducted in late 2009. It included a quantitative survey with 330 ART prescribed patients from ten ART sites across Nepal and qualitative in-depth interviews with 34 purposively selected participants, namely 17 ART-prescribed patients, 14 ART service providers (including doctors, nurses, paramedics), and three policy-makers.

For the survey eligible respondents were taken from a daily hospital visit schedule using a systematic random sampling technique [14]. Adherence was assessed by using a self-reported Adult AIDS Clinical Trial Groups (AACTG) adherence instrument [15], originally developed in the United States and now widely adopted in many countries [16], [17]. In addition to the AACTG questionnaire, a further structured questionnaire was developed from the literature. The dependent variable was ‘adherence to ART’, independent variables were socio-demographic and cultural, health condition, and service facility, knowledge, and perception of ART treatment related variables. Patients were considered adherent when they took 100% (not missing a single dose of ART) based on self-report in a four-week recall prior to the study; otherwise, they were categorized as non-adherent. If they reported having missed or skipped a dose during that time, the questionnaire asked a range of multiple-choice questions about why they had missed their pills.

Logistic regression analysis was performed to examine the effects of explanatory ( = independent) variables on adherence. All variables, both explanatory and dependent, were coded as binary variables prior to fitting in the model; for adherence this meant that 0 = non-adherent and 1 =  adherent. Initially, explanatory variables were included in the model one at a time to examine their univariate relationship with adherence. As many variables were analysed, only those that were significant are presented. Following on from this univariate analysis, multiple logistic regressions was used to model the effects of many variables simultaneously. Variables were fitted in the model in blocks: 1) socio-demographic and cultural; 2) health condition; 3) health-service facility; 4) knowledge; and 5) perception related variables. At each stage, the least significant variable was excluded until the model contained only statistically significant factors. Statistical analyses were conducted using the Statistical Package for the Social Science (SPSS) version 17.0 and a p-value of less than 0.05 was used to define statistical significance.

The in-depth interviews were conducted with the aid of an interview checklist and probes for further questioning [14], covering questions on how ART fitted into their daily routines and what key factors were impeding their adherence to their medication. The interviews were conducted and audiotaped in Nepali by the first author who is a native speaker; sessions lasted 1–1½ hours. Audio recordings were transcribed verbatim in Nepali and then translated into English. Data were organized using NVivo version 8 [18] and coded using a thematic analysis [19]. The quotations included below best represented the range of ideas voiced around key themes; to maintain anonymity these quotes are identified by gender and region only. For the same reason, nurses and paramedics are all coded as ‘counsellor’. To assure quality of the data, the main survey questionnaire was piloted [20] with 15 selected ART prescribed patients and the semi-structured questionnaire (checklist) was piloted with two patients on ART and a care provider; subsequently some modifications were incorporated in the research instruments. Ethical permission for the study was obtained from the Nepal Health Research Council. Individual consent was sought before interviews, often in the form of oral consent as about 42% of the population in Nepal is illiterate [21].

## Results

### Quantitative Results

In total 330 ART prescribed patients were approached and 100% responded to the main questionnaire; a total of 57.0% were male and the mean age was 35.8 (SD 8.30) years (range 18 to 62). The majority (70%) of the respondents were married, 38.5% had never attended school, 83.0% were unemployed, and 25.2% of the respondents travelled more than three hours to the hospital. The majority (68.5%) had disclosed their status to someone other than the health care worker. More than a quarter (30.9%) faced some form of stigma and discrimination because of their HIV positive status. Patients had been taking ART for a median of 24 months (range 4 to 48). The first line treatment regimens based on 2NRTIs+1NNRTI (Non/Nucleoside Reverse Transcriptase Inhibitor) were used by almost all patients (99.4%). Interestingly, none of the respondents were taking any traditional medicine with ART for HIV treatment. All respondents identified the trade names, dosage, and frequency of pills and all understood the notion of adherence. The majority of the respondents had positive perceptions of ART treatment and maintaining adherence; for example, 95.2% perceived that even after feeling better, patients should not stop taking ART medication.

Adherence in this study is encouraging with 282 (85.5%) respondents reporting they had not missed a single dose in the previous four weeks. When asked why they had missed their pills, the most commonly reported reasons were transport costs (64%); pills running out (62%); not wanting other people to notice (62%); wanting to avoid side-effects (58%); and busy with other things (48%) (Table 1).

**Table 1:** Socio-demographic characteristics of the sample.

Variables	N	(%)
**Sex**		
Female	142	(43.0)
Male	188	(57.0)
**Level of education**		
Never attended school	127	(38.5)
Primary school	122	(37.0)
Secondary school (SLC)	44	(13.3)
College level and above	37	(11.2)
**Unemployed**	274	(83.0)
**Disclosed HIV status**	226	(68.5)
**Experienced stigma/discrimination**	102	(30.9)
**Use alcohol**	65	(19.7)
**Used memory aid**		
Watch/mobile alarm	249	(75.5)
Do not have any memory aids	66	(20.0)
Written schedule	12	(3.6)
Text message by health worker	3	(0.3)
**Having family support**		
No one	141	(42.7)
Spouse	130	(39.4)
Family members	51	(15.5)
Best friend	8	(2.4)
**ART initiated (months)**Median (IQR)	24.0 (12 to 36)	
**Regimens**		
First line	328	(99.4)
Second line	2	(0.6)
**Time to reach hospital**		
>1 hour	194	(58.8)
≤1 hour	136	(41.2)
**Self-reported adherence**		
Four days adherence	312	(94.5)
One week adherence	307	(93.0)
Four weeks adherence	252	(85.5)
**Reasons for missing medication (N = 48)**(*multiple responses allowed)*		
Not having travel fare	32	(64.0)
Pills ran out	31	(62.0)
Not wanting other people to notice	31	(62.0)
Wanted to avoid side-effects	29	(58.0)
Were busy with other things	24	(48.0)
Felt sick or ill	18	(36.0)
Nepal strike (transportation blocked)	17	(34.0)
Simply forgot	17	(34.0)
Were away from home	13	(26.0)
Alarm not working /slept in	8	(16.0)
Taking alcohol at specific time	5	(10.0)
Problems to take pills at specified time	5	(10.0)
Fasting	2	(4.0)
Family quarrel	1	(2.0)

### Factors Associated with Adherence to ART

The univariate analysis found that 24 variables were significantly associated with adherence (Table 2), although of these only ten remained significant in the final multivariate logistic regression model: patients who had not disclosed HIV status to anyone other than health workers (OR = 17.99, p =  0.014), who drank alcohol (OR = 12.89, p = <0.001), were female (OR =  6.91, p =  0.001), illiterate (OR =  4.58, p =  0.015), had a regimen side-effect (OR = 6.04, p = 0.025), duration of ART started ≤ 24 months (OR = 3.18, p =  0.009), travelled more than 1 hour to hospital (OR =  2.84, p =  0.035) were significantly more likely to be non-adherent. Similarly, patients who thought that HIV would disappear after ART (OR = 6.82, p = 0.008), that distance affects ART adherence (OR  = 15.33, p =  0.008), that you could stop taking the medicine once you felt better (OR =  6.43, p = 0.011), or that HIV was preventable by taking regular ART (OR = 4.31, p = 0.003) were significantly more likely to be non-adherent.

**Table 2:** Potential factors influencing adherence to ART.

Variables	AdhereN (%)		Non-adhereN (%)		UnivariateOR	p	MultivariateOR	p
**Alcohol intake**								
Yes	40	(61.5)	25	(38.5)	6.58 (3.41 to 2.69)	< 0.001	12.89 (3.97 to 41.85)	<0.001
No	242	(91.3)	23	(8.7)				
**Drug side-effects**								
Yes	195	(81.9)	43	(18.1)	3.84 (1.47 to10.02)	0.002	6.04 (1.25 to29.08)	0.025
No	87	(94.6)	5	(5.4)				
**Occupation**								
Unemployed	229	(83.6)	45	(16.4)	3.47 (1.04 to 11.60)	0.043		
Employed	53	(94.6)	3	(5.4)				
**Distance to travel hospital**								
> one hours	159	(82.0)	35	(18.0)	2.60 (1.27 to 5.31)	0.009	2.84 (1.08 to 7.49)	0.035
≤ one hours	123	(90.4)	13	(9.6)				
**Missing schedule visit**								
Yes	139	(80.3)	34	(19.7)	2.50 (1.29 to 4.86)	0.005		
No	143	(91.1)	14	(8.9)				
**Means to travel hospital**								
On foot	64	(76.2)	20	(23.8)	2.43 (1.29 to 4.61)	0.006		
By vehicle	218	(88.6)	28	(11.4)				
**Duration of ART started**								
≤24 months	162	(81.8)	36	(18.2)	2.22 (1.11 to 4.45)	0.024	3.18 (1.34 to 7.55)	0.009
>24 months	120	(90.9)	12	(9.1)				
**Education**								
Illiterate	100	(78.7)	27	(21.3)	2.34 (1.26 to 4.35)	0.007	4.58 (1.34 to 15.71)	0.015
Literate	182	(89.7)	21	(10.3)				
**Age**								
≤ 35 years	152	(81.7)	34	(18.3)	2.08 (1.07 to 4.04)	0.031		
>35 years	30	(90.3)	14	(9.7)				
**Number of pills per day**								
> Three tablets	126	(80.8)	30	(19.2)	2.06 (1.09 to 3.87)	0.024		
≤ Three tablets	156	(89.7)	18	(10.3)				
**Disclosure of status**								
No	82	(78.8)	22	(21.2)	2.06 (1.11 to 3.85)	0.023	17.99 (1.81 to 78.48)	0.014
Yes	200	(88.5)	26	(11.5)				
**Gender**								
Female	114	(80.3)	28	(19.7)	2.06 (1.06 to 4.06)	0.022	6.91 (2.31 to 20.67)	0.001
Male	168	(89.4)	20	(10.6)				
**Stigma and discrimination**								
Yes	81	(79.4)	21	(20.6)	1.93 (1.03 to 3.61)	0.039		
No	201	(88.2)	27	(11.8)				
**Knowledge on forgetting ART cause problems**								
No/don’t know	6	(54.5)	5	(45.5)	5.35 (1.56 to 18.29)	0.014		
Yes	276	(86.5)	43	(13.5)				
**HIV will disappear after ART**								
Yes/don’t know	5	(55.6)	4	(44.4)	5.04 (1.31 to 20.95)	0.027	6.82 (1.64 to 28.31)	0.008
No	277	(86.3)	41	(13.7)				
**ART cause side-effects**								
Yes	193	(82.1)	42	(17.9)	3.23 (1.32 to 7.87)	0.005		
No/don’t know	89	(93.7)	6	(6.3)				
**Perception that if patients feel well, they can stop taking their ART**								
Yes/don’t know	7	(43.8)	9	(56.3)	9.07 (3.20 to 25.73)	<0.001	6.43 (1.53 to27.04)	0.011
No	275	(87.6)	39	(12.4)				
**Distance affects ART adherence**								
Yes	232	(83.5)	46	(16.5)	4.96 (1.17 to 21.09)	0.010	15.33 (2.06 to 14.25)	0.008
No/don’t know	50	(96.2)	2	(3.8)				
**ART recipients will get sicker if they stop taking their medication**								
No/don’t know	13	(59.1)	9	(40.9)	4.78 (1.92 to 11.91)	0.001		
Yes	269	(87.3)	39	(12.7)				
**ART prevents HIV/AIDS disease progression**								
No/don’t know	27	(64.3)	15	(37.5)	4.29 (2.07 to 8.89)	0.015		
Yes	255	(88.5)	33	(11.5)				
**ART cure on HIV**								
Yes/don’t know	11	(61.1)	7	(38.9)	4.21 (1.54 to 11.47)	0.009		
No	271	(86.9)	41	(13.1)				
**ART must continue even after feeling better**								
No/don’t know	8	(61.5)	5	(38.5)	3.98 (1.25 to 12.74)	0.033		
Yes	274	(86.4)	43	(13.6)				
**ART should not stop after gaining weight**								
No/don’t know	9	(64.3)	5	(35.7)	3.53 (1.13 to 11.02)	0.046		
Yes	273	(86.4)	43	(13.6)				
**HIV is preventable by taking regular ART**								
Yes/don’t know	56	(76.7)	17	(23.3)	2.21 (1.14 to 4.28)	0.022	4.31 (1.67 to 11.13)	0.003
No	226	(87.9)	31	(12.1)				

The following were significant in the univariate analyses but were not found to be so in the multivariate analysis: being unemployed, missing scheduled hospital visit, method of travelling to hospital, age ≤ 35 years, number of pills taken per day, feeling stigmatised or discriminated against, knowing that ART caused problems and side-effects, and that treatment needs to be continued even when feeling better or weight has been gained. There was also no significant difference in the multivariate analyses between those patients who did or did not know that ART prevents HIV/AIDS progression; that ART did not cure AIDS; and that forgetting ART can cause problems.

#### Qualitative results

The results of the in-depth interviews complemented the survey and shed further light on its findings. The thematic analysis suggested a range of factors which negatively influenced adherence to ART.

### Perception About ART

Perception is concerned with people’s beliefs that they can exert control over their own motivation, thought processes, emotional states and patterns of behaviour. However, negative perceptions whether the efficacy of ART and its effects and could act as barriers and be preventing adherence. For example, one participant discussed that:

*Rural people do still not believe this medicine [ART] work for HIV patients. HIV people will die eventually either taking or not taking ART. Why should I die by taking these malicious pills? They stopped taking medicine after initiating treatment (P- 12, Female, Far-western).*

### Religion and Rituals Obstacles

People live in a community and need to abide by their local traditional and religious rituals, which can influence adherence to ART. For example, a Muslim reported:

*I stopped my morning ART during Ramadan …I was sick and went to consult the doctor and he told me not to stop at any time….now I am taking medicine when fasting (P - 16, Female, Highway).*

Health care providers noticed that some PLHIV did not take their morning ART because their culture required fasting from sunrise and to sunset.

*Some Muslim patients have changed their [ART] routine due to their festival and they left out the morning dose. This is due to their culture (P - 25, Counsellor, Highway).*

A doctor also stated that in both Hindu and Muslim festivals women fast adding:

*I am not blaming all my patients but a few Hindu women during Teej and Muslim patients in Ramadan have problems taking medicine … a few Muslim patients did not take medicine in the morning because of Ramadan. They told me if they drink water during the day this is totally sinful (P - 27, Doctor, Kathmandu).*

Religious constraints would seem to remain the most significant barrier to adherence and where this is compounded by unsympathetic family circumstances as discussed below, non-adherence may be even more likely to occur.

### Alcohol Intake

Alcohol intake was mentioned by PLHIV as contributing to non-adherence. Some stated that at festival times they were expected to drink alcohol and that this caused difficulty with remembering their medication routine. A male disclosed that:

*I need to attend parties and drink during festival times, when I drink alcohol; I have great difficult in taking my night dose [of ART]. I missed it many times (P - 13, Male, Hill).*

Health care providers also agreed that some patients had reported missing ART due to alcohol consumption.

### Lack of family support

Lack of family support acted as a barrier to adherence and family arguments stopped them from taking medication, as one female participant explained:

*I skipped two doses because of a family quarrel my family did not allow me to visit hospital (P - 14, Female, Highway).*

Some professionals also noted that patients without family support had lower adherence levels, e.g.:

*One patient … skipped medicine due to no one getting her medicine from the hospital for her. She was bed-ridden, but family members did not bring medicine. Because the pills ran-out and no one helped her, she skipped some doses [of ART] (P - 18, Counsellor, Far-western).*

Conversely, one female participant narrated how her family helped her,

*…I often forgot to take ART, my daughter ask me, mummy, have you taken your medicine today? …. She reminds me. Almost all the time she [daughter] brings a glass of water and pack of medicines (P - 12, Female, Far-western).*

Thus, the combination of difficulty of accessing hospital and an unhelpful family can negatively affect adherence whereas family support enhance adherence.

### Economic Constraints

Money emerged as the most commonly mentioned barrier to adherence, as most respondents reported economic worries related to the cost of (a) transport, (b) prescription, (c) diagnosis, and (d) food. Transport costs emerged as a key theme and PLHIV often did not have enough money to go to the health facility to get their repeat prescription (‘refill’). One interviewee reported;

*… I missed my medication twice because of money. I need to find 600 rupees (∼£5) for bus fare every month to refill ART. How can I afford this [cost]? I do not have any alternative except missed treatment (P – 2, Male, Highway).*

Health care providers also concurred with this. This may remain a major reason why some individuals may not be able to come to the health facility to refill their prescriptions.

### Stigma and Discrimination

Most participants had experienced some form of discrimination, and HIV-related stigma (or fear of stigma) was identified by the majority of interviewees as influencing adherence behaviour especially among women. For example:

*I have not taken my medication two occasions because relatives and neighbours were in my house. I did not get time to take out medicine from my drawer…… I have frequent problem to hide my medicine from others because I am living in a rented single room. ….Oh! I cannot tell anyone (P - 5, Female, Kathmandu).*

Due to the fear of being victimised and/or rejected by their family or community, this generated a fear of exposure, which in itself affected adherence, e.g.:

*I am worried about meeting my neighbours in hospital for refills [ART]. All the time I worry: “How can I hide from these people?” One day I did not refill my ART due to bumping into relatives (P - 4, Female, Kathmandu).*

Some care providers noted that PLHIV selected ART sites where no one would know them:

*Many patients still prefer to visit the distant hospital; they are not ready to go nearby. This all about the fear of [disclosure to] other people (P - 20, Counsellor, Kathmandu).*

A doctor added:

*Clients are still stigmatized …. they fear other people …’What do they think?’, ‘What do they say?’ clients are not ready to take medicine in front of other people …they choose the alternative and skip medication (P – 27, Doctor, Kathmandu).*

### Distance

Nepal’s large rural hinterland combined with a limited number of ART sites was perceived to have a negative effect on adherence. Distance was a big concern particularly outside Kathmandu. Long travelling distance to and from ART sites remains one of the most challenging adherence issues and it was frequently discussed by both PLHIV and providers:

*I need to walk more than one and half days to refill my medicine [ART] …this is very difficult at my age, not once a year, but every month (P - 1, Male, Highway).*

Staffs also considered distance to be a big hurdle to adherence, as one counsellor discussed,

*[PLHIV walk to] … this hospital is more than two days on foot. Oh! Some clients have to cross so many small and medium rivers to come to the hospital. No bridge. I do not think it is fair to blame them for defaulting (P - 18, Counsellor, Far-western).*

Similarly, policy-makers agreed that distance and centralised ART-providing institutions limited adherence:

*Treatment services are located in a limited number of central hospitals, which is a major problem for HIV positive people seeking services. That’s definitely limiting their adherence HIV treatment service as is still not reaching the people in need (P - 33, Policy level).*

Thus the poor, and rural dwellers, who have difficulty travelling long distances for ART, may benefit from nearby ART facilities. Although this may be offset by the fear of disclosure, thus causing them to continue to travel to more distant sites.

### Short Period of Medicine Prescription

Having few ART institutions is itself a barrier to getting repeat prescriptions on time as well as different prescribing policies in different hospitals. Some clinicians prescribe ART drugs for one month, others up a maximum of two months. ART prescription practice is not associated with a single issue but it was linked with multiple factors i.e. cost, transport facilities, time. Some interviewees stated that;

*Doctors prescribed ART only for a month. This is very short time….every month to refill ART is extremely difficult to adhere this medication (P - 5, Female, Kathmandu).*

However, rural clinicians mostly prescribed up to two months of ART, for example:

*I have to visit this clinic every two months to refill medicine. I have to arrange money and need to walk long distance which is very hard; not only for me but most of the PLHIV [from hill districts]. To refill ART at every two months is the main problem for me sir (P - 10, Male, Hill).*

### Insufficient Pills in Shell Pack Bottles

There can be a discrepancy between the labelling and the contents of packages. Insufficient number of pills in a bottle or pack was raised as a barrier to adherence. Sometimes there were fewer pills in a pack than stated on the label. A health care provider reported that some patients had been supplied with an inadequate number of pills and hence missed one or two doses.

*One patient complained that the label seal packed bottle did not contain the complete number of pills according to label ( = 60) he complained to me that there were just 58 pills and he did not get one dose according to the schedule of ART refill (P - 19, Counsellor, Hill).*

Counting pills in front of patient may be necessary to ensure that the patient is given the full dose of medicines prescribed and would avoid incomplete supplies.

### Strikes (Transportation Blocked)

Nepal’s political situation was very volatile at the time of the study, which led to frequent and unpredictable strikes. This often meant that main roads were blocked and this obstacle to transport impeded ART adherence, for example:

*Unexpected strikes are in fashion at the moment….bus strikes, and last time even the hospital was on strike for a week…. How can we take regular medicine? … ‘Think about Nepal’s geography and distance, how can all ART recipients receive ART medicine without missing any?’ (P - 5, Female, Kathmandu).*

Service providers also agreed that, due to frequent and unexpected roadblocks, PLHIV had no alternative than to stop medication or miss some doses of their medicine.

### Side-effects of ART

ART side-effects were one of the most discussed themes in this study. Most participants had experienced side-effects, which increased non-adherence, e.g.:

*ART caused a problem rather than good health. I could not tolerate the pain and I stopped for a while (P - 9, Male, Highway).*

Similarly, a female PLHIV found that ART:

*… has given more side-effects for me such as vomiting, herpes/ zoster and skin rashes. I have lost my sight in my right eye and my left eye also has poor vision (P - 12, Female, Far-western).*

Doctors agreed that side-effects led some patients to stop taking ART. These common side-effects included vomiting, diarrhoea, body pain, skin rashes and reduced body fat.

### Gender and Adherence

Due to socio-cultural and economic restrictions put upon women in Nepal, women found it far more difficult to adhere to their ART medication than their male counterparts did. Most of the health-care providers mentioned that men had better adherence than women did. A care provider argued that:

*Male adherence is better, they [male] do not need to be accompanied to refill prescription, they can travel easily, most of them disclosed [HIV], they can easily understand, whereas women had more difficulties in understanding. Also treatment usually start when they (women) were bedridden (P - 22, Counsellor, Highway).*

## Discussion

The rate of adherence in our sample was significantly higher (85.5%) than has been observed in other studies in Asian developing countries that also relied upon self-report [22]–[24]. A meta-analysis found that patients in Africa had better adherence than their North-American counterparts with pooled estimates of 77% of participants achieving adherence in the African studies and 55% of participants in the North-American studies [9]. The possible explanation for greater adherence in our study might be due to using (a) self-reported adherence; (b) only first-line regimens; (c) strict adherence counselling sessions pre and post ART; and (d) recruiting participants through clinics so that only active patients participated (patients who actually went to the clinic).

Many factors identified in this study are consistent with the literature from both developed and developing countries. In this study, females reported poorer adherence than males as corroborated by care providers. This finding is unique since very few studies have reported that men were more likely to adhere to ART than women [25]–[27]. This is an important finding in the Nepalese context, as women are treated differently and unequally due to socio-cultural and economic factors. Nepalese women are recognized to be ill by family members only when they are bedridden and the head of the household (usually their husband or mother-in-law) decides that they are seriously ill. Women in this study were less likely to disclose their HIV status (66.2% vs 70.2), and were more vulnerable to discrimination by others compared to male PLHIV (45.1% vs 20.2%), both of which could be used to explain this finding. Different forms and types of HIV-related stigma exist such as self-stigma (self-blame or condemnation), perceived stigma (fear of stigma associated with disclosing HIV status) and enacted stigma (gossip, rejection, discrimination) and these were barriers to adherence [28], [29]. Our qualitative findings also revealed that stigma (or fear of stigma) was identified by the majority of interviewees as impeding adherence behaviour particularly among women. This will become an increasing problem because of increasing female infection rates in Nepal, but currently the Government still lacks programmes targeted to test, treat and improve treatment adherence for women [30]. Nepali women require special and urgent consideration because of their socio-cultural and sexual subordination [31], [32]. Additionally, women are most often responsible for care of the household and children, and as a consequence these practical barriers may adversely affect adherence among women more than among men [33]. Thus, interventions that focus on women taking ART may benefit from paying special attention to the multiple life demands that can interfere with their ability to refill medication or obey specific instructions from their care provider.

Other adherence barriers raised during the interviews included local culture, especially religious activities and festivals such as “Teej” for Hindu women and “Ramadan” for Muslims. Religious beliefs are complex concepts and are part of the basic assumptions which shape people’s identities and strongly affect their decision-making such as taking medication on fasting days [34], [35]. It is difficult to see how this could be overcome but our findings reinforce the importance of considering the religious and spiritual beliefs of PLHIV as part of medical care. It has been suggested that incorporating discussions about spiritual beliefs into adherence counselling could foster adherence [36]. As a result, patients could be motivated by seeing improvements in their health condition to continue adherence all the time. It is believed that most religions give freedom to eat on fasting day especially to the sick, children, and older people. This message needs to be reinforced during counselling.

Similarly, being illiterate, drinking alcohol and starting ART within the past two years were all associated with increased likelihood of non-adherence to ART in our study and similar findings have been reported elsewhere [25], [37]–[41]. Education may impact on adherence in several ways including facilitating communication with health care providers, increasing retention of information provided by health workers and thereby enhancing adherence to ART medication. It is possible that patients with limited literacy might be reluctant to ask others for the kind of help they need to take their medicines correctly [42]. Better-educated people convinced of ART efficacy, perhaps as a result of educational programmes, show a propensity towards better adherence [43]. A meta-analysis of drinking alcohol showed that individuals who drank alcohol had a reduced adherence to ART of between 40% to 50% [40]. Therefore, adherence interventions among females, those new to ART, illiterate patients and patients who drink alcohol need to consider these factors when reinforcing continuing adherence practices [33], [44]. It may be necessary to schedule follow-ups that are more frequent, monitor adherence more closely and ensure that education and discussions take place in a safe environment.

Patients’ beliefs, knowledge and expectations regarding treatment strongly influence their medical decision making [45]. Our findings show that a few patients believed that ART cured HIV or were unclear about how long they should take ART. Every ART-prescribed patient needs to understand that with appropriate treatment and continuing adherence HIV/AIDS is now a manageable chronic disease [2], [46]. Therefore, every patient needs to understand the importance of adherence because inaccurate information and misconceptions towards the disease and treatment regimens are associated with poor therapeutic outcomes [47] that in turn may be an impediment to achieving optimal levels of adherence [33], [48]. PLHIV who believe in the efficacy of ART are more likely to adhere [49]. Hence, care providers should continue to educate PLHIV and their families and develop intervention strategies that address the local context in order to encourage people to adhere to ART.

Although ART drugs have been provided free of charge since 2004 in Nepal, ART associated costs are not (including transport, prescription, procuring food and diagnostic testing) and remain barriers. In addition, 62% of respondents cited pills running out as a second main reason for skipped medication. These ART-associated costs have been found to be a barrier to adherence in other Asian developing countries [13] and many other developing countries [9], [50]–[52]. A study in India found that almost all the participants discussed the cost of ART as a barrier, with many reporting drug holidays, turning to family and or friends or taking drastic measures (i.e. selling jewels, property) for financial assistance [53]. A study in Botswana showed that adherence was higher when cost was not an obstacle and poor patients were able to achieve excellent rates of adherence when they had access to free laboratory monitoring and subsidized ART [54]. This is a useful finding and it is important that policy makers use this information to introduce social policy which includes subsidies for transport, and other costs associated with ART. Addressing the ART-associated cost issue of non-adherence in Nepal may therefore require a somewhat different approach to solutions applied in developed countries where financial issues are not such a major concern.

In the in-depth interviews, participants raised the issue that physicians prescribed ART for periods that were too short thus increasing costs associated with transportation to collect medication. This has also been documented elsewhere [55], [56]. Patients were worried and reported that to refill medicines every month caused difficulties in maintaining adherence. Thus, the Government of Nepal needs to consider carefully the amount of medicine that is prescribed on each occasion as well as subsidizing travel costs. To make available ART collection points in local health institutions may also help to manage this short period between refills. Once these have been implemented, further research will need to be focused on the effectiveness of these and other possible interventions to decrease barriers related to transportation costs.

In addition, non-disclosure of HIV status to anyone except health workers was associated with poorer adherence in our study and has been documented elsewhere too [9], [13], [57], [58]. Studies have shown that HIV disclosure is necessary to facilitate adherence to ART [59] and PLHIV who did not disclose to their family or peers had poorer adherence than those who disclosed their status [9], [59]. Our findings suggest that disclosure is an important issue, which should be discussed during the counselling sessions. For those who have not disclosed, providers should explain the importance of disclosure for the success of ART.

Skipping and stopping doses were attributed to fear that other people would discover their HIV status. Stigma and discrimination were not found to be significant influencing factors in the multivariate analysis, but in the in-depth interviews the majority of participants (seven female vs. four male) raised as this as a barrier to adherence. Patients were too embarrassed to take their medication in front of others, and concerned about privacy when collecting repeat prescriptions and taking medication; these worries increased non-adherence and have also been found in other studies outside Nepal [13], [58], [60]. One study stated that PLHIV were unwilling to seek treatment at the nearest health facility because of fear of stigmatization [61] and this same issue was discussed in our study too. Hence, policy makers should encourage a supportive environment where PLHIV do not need to worry about stigma and discrimination but talk openly in order to facilitate adherence. In addition, patients should also be taught strategies on how to handle taking pills in secret to increase adherence to their medication.

Distance to treatment centre is of great concern to PLHIV and one of the key factors preventing adherence in this sample. Patients who travelled more than one hour to hospital were more likely to be non-adherent and this was also discussed in the in-depth interviews. Participants stated that although patients were willing to take ART they became non-adherent because of difficulties in reaching the treatment centres due to unexpected transport and other strikes; long travel distance; geographical difficulty including lack of transportation services in many remote areas; and the seasonal deterioration of poorer roads during the rainy season. This has also been found to be the case with respect to maternity services in Nepal [62]. Others have also found that travel time and access to treatment centres were barriers to ART adherence [52], [63] and that better access to care was significantly associated with optimal adherence [13]. Patients who are from rural areas have difficulties in travelling long distances and finding their travel costs, and have most to gain from nearby ART facilities. Although this benefit may be offset by patients who fear of disclosure and worry about others finding out, are still travelling to more distant sites. Thus, any new policy will need to address this issue and improve access to medical care services by integrating ART treatment into the mainstream of health care rather than concentrating treatment in a limited number of ART centres, which may be hard to reach for many patients.

ART regimens have toxicities and adverse side-effects that vary from mild to severe and acute to chronic, which can prevent adherence [64]. Our study revealed that patients who had side-effects were more likely to be non-adherent and this has also been reported from several studies conducted in both developed and developing countries [9], [13], [65]. Similarly, patients who have been on ART for less than two years were more likely to be non-adherent, but there is no clear indication for why this is the case. There may not be a single reason behind non-adherence of this group but side-effects may be one of the possible explanation because the literature shows that up to 45% of ART prescribed patients discontinue one or more of the drugs within a year of starting due to side-effects and the inconvenience of the regimen [66]. Therefore, there is a need for continuing follow-up, clinical support and counselling for patients on ART in the early years of treatment as well as providing instructions on how to cope with side-effects [67]. Health care providers should review possible anticipated side-effects and develop treatment plans that either prevent, or at the very least, reduce the likelihood of side-effects happening. These may be tackled by strengthening institutional support, including clinicians and other health professionals informing patients in advance what side-effects to expect and how they might be managed, as well as prescribing antiemetic or anti-diarrheal agents to alleviate these side-effects. In addition interviewees raised the issue of discrepancies between the labelling and contents of packages of pills. Therefore, counting pills in front of patients is necessary to ensure complete dose of medicines prescribed to avoid incomplete supplies.

Participants discussed the twofold nature of social support systems: lack of support as a barrier to adherence and, when actually in place, as a facilitator of adherence [68]. Some people were accepting and supportive whilst others overtly or subtly distanced themselves from people with HIV. In this study, family members reminded 57.3% respondents of their medication although this was not significantly associated with adherence. Furthermore, findings from the in-depth interviews highlighted that family support increased the likelihood of patients maintaining optimal adherence. Others have also found that social and family support, communication and access to care that anticipate patients’ individual needs play an important positive role [9], [13], [69], and family acts as a facilitating factor of adherence primarily among children and women [70]. A meta-analysis reported that adherence is 1.74 times higher in patients from cohesive families [71] whereas absence of this is a factor leading to poor adherence [72]. Therefore, health care providers should continue discussions with new starters and follow up individuals without support to find an alternative source of help to foster adherence.

### Strengths and Limitations of the Study

The main strength of our study is that it is the first to examine adherence to ART in Nepal. Using a mixed-methods approach across multiple sites allowed for triangulation through synthesising data from multiple sources of informants. Mixed methods assisted in highlighting important factors, which may not be effectively explored by using only a single method. Our study had an extremely high response rate. No one who was asked to participate refused to do so. We think this is because HIV research is in its infancy in Nepal and this was the first ever research project for this population of PLHIV. Moreover, it offered an opportunity for PLHIV to talk about living with HIV in a society where this is generally problematic.

However, the study does have some limitations. Due to financial constraints adherence was assessed through a self-reporting adherence questionnaire and not other more objective tools such as electronic pill caps, pills counts, and biological methods (patients’ viral load and CD4 count) that should be priorities for future studies. Despite concerns that self-reporting may overestimate adherence, it has been demonstrated elsewhere that self-reported adherence has consistently correlated with viral load and clinical outcomes in HIV treatment [73]–[75], as well as improved quality of life [74], and has been deemed a robust and appropriate indicator of adherence [76].

The second major limitation of this study is that the respondents consisted only of patients who actually went to ART clinics. We did not interview people who were picking up ART drugs for someone else and this means that we may have missed patients too sick to attend appointments. Additionally, those who defaulted from the ART service were not interviewed. Thus our participants were motivated and well enough to attend ART clinics, this may also explain why all could say at least the trade names of the ART they were taking. We would recommend that future researchers should try to trace defaulting ART patients and identify challenges perceived by these patients.

This study is a cross-sectional study, which measured adherence at a single time point. However, adherence is a dynamic process that may change over time; thus, it may be that multiple contacts with respondents could have provided more useful information than a single interview. Therefore, longitudinal studies with a wider range of respondents and the use of a combination of adherence assessment tools are also necessary in this setting to understand adherence over time and to explore the factors that influence adherence to ART in the longer term, which could most likely reduce the risk of overestimation.

### Conclusion

Adherence (85.5%) in Nepal is sub-optimal (defined as less than 95%) but this finding is similar to other Asian developing countries, as well as being better than has been measured by self-report in many developed counties. There are a range of reasons for failing to adhere to ART, including drinking alcohol, having drug side-effects, long distance to travel to hospital, being illiterate, non-disclosure of HIV status, being female, lack of knowledge and negative perceptions towards ART. The key reason for skipping ART given was travel fare problems presumably in order to collect ART, followed by pills running out and wanting to avoid the side-effects. Qualitative findings also added that religious or ritual obstacles, embarrassment about taking medication in front of others, financial constraints, and transport problems including strikes and adverse side-effects were important factors in non-adherence. Priorities should be given to improving adherence by providing regular follow-up, increasing patients’ awareness of the ART treatment, including its benefits and side-effects, eliminating problems of access and alleviating the impact of cost. Policy makers need to be aware of these key barriers and consider social policy which encourages patients to achieve optimal adherence.
